# Deciphering the connection between upstream obstacles, wake structures, and root signals in seal whisker array sensing using interpretable neural networks

**DOI:** 10.3389/frobt.2023.1231715

**Published:** 2023-08-03

**Authors:** Dariush Bodaghi, Yuxing Wang, Geng Liu, Dongfang Liu, Qian Xue, Xudong Zheng

**Affiliations:** ^1^ Department of Mechanical Engineering, University of Maine, Orono, ME, United States; ^2^ Department of Computer Engineering, Rochester Institute of Technology, Rochester, NY, United States; ^3^ Department of Engineering, King’s College, Wilkes-Barre, PA, United States; ^4^ Department of Mechanical Engineering, Rochester Institute of Technology, Rochester, NY, United States

**Keywords:** bioinspired flow sensing, wake identification, seal whisker, interpretable machine learning, fluid-structure interaction

## Abstract

This study presents a novel method that combines a computational fluid-structure interaction model with an interpretable deep-learning model to explore the fundamental mechanisms of seal whisker sensing. By establishing connections between crucial signal patterns, flow characteristics, and attributes of upstream obstacles, the method has the potential to enhance our understanding of the intricate sensing mechanisms. The effectiveness of the method is demonstrated through its accurate prediction of the location and orientation of a circular plate placed in front of seal whisker arrays. The model also generates temporal and spatial importance values of the signals, enabling the identification of significant temporal-spatial signal patterns crucial for the network’s predictions. These signal patterns are further correlated with flow structures, allowing for the identification of important flow features relevant for accurate prediction. The study provides insights into seal whiskers’ perception of complex underwater environments, inspiring advancements in underwater sensing technologies.

## 1 Introduction

Given the limitations of the current underwater navigation systems, there has been a growing interest in advancing underwater sensing capabilities. The increasing scientific, military, and commercial demands, along with the exploration of the uncharted underwater world, have been the driving force behind these developments. Underwater robots employ various navigation systems, including inertial, geophysical, and acoustic systems, to estimate their position, velocity, and acceleration (Stutters et al., 2008; Chutia et al., 2017). However, these systems rely heavily on seafloor maps to prevent collisions and have limited capabilitiesin surveilling, navigating, and maneuvering safely in unpredictable deep waters, while avoiding obstacles and hazardous environments (Kinsey et al., 2006; Leonard and Bahr, 2016).

To address these challenges, different types of sensors are utilized on underwater robots (Chutia et al., 2017; Elshalakani et al., 2020). Camera-based sensors, relying on vision, are commonly used for monitoring the nearby environment and object detection. However, their effectiveness is much lower in dark deep waters, necessitating the use of artificial light sources. This further reduces the sensor’s range and reveals its location, posing a significant disadvantage, particularly in military applications (Yang et al., 2006; Jiang et al. 2019; Jiang et al. 2020). In environments with poor water clarity, the images captured by these sensors suffer from low quality and cloudiness due to light dissipation (scattering) over long distances (Kröger, 2008; Lee et al., 2012). To overcome the limitations of vision-based sensors, sonar sensors are commonly employed to estimate object locations by transmitting acoustic waves (Akyildiz et al., 2004). However, similar to vision sensors, sonar sensors have drawbacks such as high-power consumption and the risk of revealing the sensor’s location (Akyildiz et al., 2004; Elshalakani et al., 2020). Furthermore, the use of sonar sensors can have detrimental effects on various underwater species by altering their natural habitats (Schrope, 2002).

In recent years, there has been significant research on exploring the sensing abilities of phocid seals to address challenges in underwater sensing. These seals possess remarkable whisker arrays that enable them to detect and distinguish hydrodynamic disturbances caused by potential prey. Studies have shown that seals can detect disturbances with speeds as low as 245 μm/s and frequencies ranging from 10 to 100 Hz (Dehnhardt et al., 1998). Behavior studies have demonstrated the seals’ capability to discern the shape and size of upstream objects, track the travel direction of vortex rings, detect miniature submarines, and even follow other seals, by only using their whisker arrays (Dehnhardt et al., 2001; Müller and Kuc, 2007; Wieskotten et al., 2010; 2011). Notably, seals can track objects at considerable distances and detect weak vortices from past movements (Müller and Kuc, 2007). These findings highlight the extraordinary sensitivity, accuracy, and intelligence of seal whisker arrays in detecting hydrodynamic cues. The remarkable sensing abilities of seal whiskers have sparked great interest and inspiration for applications in underwater navigation, object tracking, and object detection. Several biomimetic sensors has already been designed, fabricated and tested in the laboratories (Beam et al., 2013; Kottapalli et al., 2015; Eberhardt et al., 2016; Sayegh et al., 2022; Wang et al., 2022; Zheng et al., 2022; Liu et al., 2023).

Previous studies on the fundamental mechanism of seal whisker sensing have mostly focused on the geometric effects, revealing that the whisker’s unique undulated shape can suppress self-induced vibrations by disrupting the Kármán vortex street in the wake (Hanke et al., 2010; Hans et al., 2013; Beem, 2015; Kottapalli et al., 2015; Morrison et al., 2016). Our recent study (Liu et al., 2019b) further elucidated that the suppression of the vibration is primarily due to the 180° phase shift of the axes of the elliptical cross-sections of seal whisker, which generates stable three-dimensional hair-pin vortices in the wake. Additionally, Beem and Triantafyllou, (2015) found that seal whisker geometry is able to generate large-amplitude “slaloming” motions in vortical flows by extracting energy from passing vortices, thereby enhancing wake capturing sensitivity.

While these findings confirm the high sensitivity of seal whisker sensing, the fundamental mechanism of wake identification and tracking remains largely unknown. Seal whiskers are arranged in a stereotyped grid pattern known as the vibrissa array, and the resulting bend moments at the root of the whiskers collectively form sensory inputs. Recent studies have suggested that seals may correlate temporal-spatial patterns of whisker array signals with surrounding flow patterns for intelligent perception. Various flow features, such as highest velocities, velocity gradients, and wake spatial extension, have been proposed as potential cues for object perception (Wieskotten et al., 2011). Artificial whisker array experiments have also indicated that cross-correlation of bending signals can be used to detect vortices (Glick et al., 2021). Our previous computational studies have shown that whisker array signals can reflect the strength, timing, and trajectories of upstream vortex-induced jets (Liu et al., 2021). Machine-learning models have been used to infer the position of upstream objects based on whisker tip displacements (Elshalakani et al., 2020). However, many questions remain unanswered, including the correlation between mechanical whisker signals and external flow disturbances, the specific flow features seals can sense, and how they interpret flow features to perceive the environment. Further research is needed to unravel these mysteries and advance our understanding of seal whisker sensing.

In this study, we aim to develop a novel method that combines a computational fluid-structure interaction (FSI) model with an interpretable deep-learning model to investigate the fundamental mechanisms underlying seal whisker sensing. The novelty of the method lies in its ability to establish connections between crucial signal patterns, flow characteristics, and attributes of upstream obstacles, thereby enhancing our understanding of the intricate sensing mechanisms. The effectiveness of the method is demonstrated through its ability to accurately predict the location and orientation of a circular plate positioned in front of seal whisker arrays. To achieve this, we placed a circular plate within a free stream, located upstream of the seal’s head. Two whisker arrays were integrated onto the seal’s head, replicating the realistic location, structure, geometry, and orientation observed in reported data. A one-way FSI model was employed to compute the plate-induced wake and subsequent dynamics and signals of the whiskers, represented by the root bending moment. By systematically varying the plate’s location and orientation, we generated diverse wake characteristics and whisker signals.

We further developed and trained an interpretable neural network model to learn and accurately predict the location and orientation of the upstream plate based on the whisker signals. Importantly, the model produces temporal and spatial importance values, allowing for the identification of crucial temporal-spatial signal patterns contributing to the network’s predictions. We then correlated these patterns with flow structures, enabling the identification of important flow features for prediction. The outcomes of this study provide novel insights into seal whiskers’ ability of perceiving and interpreting complex underwater environments, which have the potential to advance the development of underwater sensing technologies.

The rest of the paper is organized as follows: Section 2 introduces the computational model including the governing equations and numerical method, whisker array model, and parametric simulations setup. Section 3 introduces the interpretable deep learning network, including the network architecture, input preparation and training configuration. Section 4 presents the results of parametric simulations and network prediction and interpretation as well as the connection between signals and flow features. Section 4 presents the summary of the results and general conclusion.

## 2 Computational model

### 2.1 Governing equations and numerical method

The flow field is modeled using unsteady incompressible Navier-Stokes equations:

$$\;\frac{\partial u_{i}}{\partial x_{i}}=0$$

$$\frac{\partial u_{i}}{\partial t}+\frac{\partial u_{i}u_{j}}{\partial x_{j}}=-\frac{\partial p}{\partial x_{i}}+\frac{1}{Re}\frac{\partial^{2}u_{i}}{\partial x_{j}\partial x_{j}}$$

where $u_{i}$ represent the velocity components in the three directions and p is pressure. Re is the Reynolds number, defined as $\frac{U_{\infty }}{\nu }$ , where $U_{\infty }$ is the incoming flow speed, D is the characteristic length, and ν is the kinetic viscosity of water at 20°*C*. The presence of the whiskers in the flow field is ignored in this study.

The current study employs an in-house immersed boundary method based incompressible flow solver, as described by Mittal et al. (2008). The solver discretizes the spatial terms using a cell-centered collocated arrangement of the primitive variables $u_{i}$ and p . To integrate the equations in time, a three sub-steps fractional step method based on Van Kan (1986) is employed. In the first sub-step, an intermediate velocity field, $u^{*}$ , is determined by solving a modified momentum equation without the pressure term. The convective terms in this equation are discretized using a second-order Adam-Bashforth scheme, while the diffusion terms are discretized using an implicit Crank-Nicholson scheme to remove the viscous stability constraints. In the second sub-step, the pressure corrections are computed by solving a pressure Poisson equation, and in the final step, the pressure field and velocity field are updated using the obtained pressure corrections. To treat the immersed boundaries, a multidirectional ghost-cell methodology is employed, resulting in both local and global second-order accuracy. The solver used in this study has been successfully applied to various biological applications, including human phonation, insect flight, fish swimming, and seal whiskers inspired flow sensing (Geng et al., 2017; Liu et al., 2018; Liu et al., 2019b; Bodaghi et al., 2021a; Bodaghi et al., 2021b; Liu et al., 2021). Details of the solver can be found in Mittal et al. (2008).

Each whisker is modeled as elastic structure and dynamics of whisker is governed by the Navier equation:

$$\frac{\partial^{2}d_{i}}{\partial t^{2}}=\frac{1}{\rho_{w}}\frac{\partial \sigma_{ij}}{\partial x_{j}}+f_{i}$$

where $d_{i}$ , $\rho_{w}$ , $\sigma_{ij}$ and $f_{i}$ are displacement vector, whisker density, stress tensor and body force vector, respectively. An in-house finite element method code is utilized to solve the equation for $d_{i}$ based on the hydrodynamic loads obtained from the flow solver. This solver was fully validated by Liu et al. (2019a) and was successfully employed for different biological simulations, including fish swimming (Liu et al., 2018) and seal whiskers (Liu et al., 2021).

Upon solving the dynamic equation at each time step, the hydrodynamic loadings along each whisker are estimated based on the local flow speeds using a simplified drag-lift model. To achieve this, each individual whisker is discretized into a series of whisker segments, each one spanning one segment. The hydrodynamic force acting on each segment is computed using $\left(F_{D},F_{L}\right)=\left(C_{D},C_{L}\right)∙0.5\rho U^{2}A_{ref}$ , where $F_{D}$ and $F_{L}$ are the drag and lateral forces along the primary and minor axis of the cross section, respectively. ρ is flow density, U is local flow speed and $A_{ref}$ is referenced cross section area. $C_{D}$ and $C_{L}$ are the drag and lateral force coefficients of the whisker segment and depend on local Reynolds number and angle of attack of the segment. We obtained these coefficients by using a tabulated function from our previous numerical parametric study. The resulting hydrodynamic loadings are then applied the centroid points of each segment, together driving the motion of the entire whisker. The details of hydrodynamic load calculation can be found in Liu et al. (2021).

### 2.2 Whisker arrays model

**FIGURE 1:**
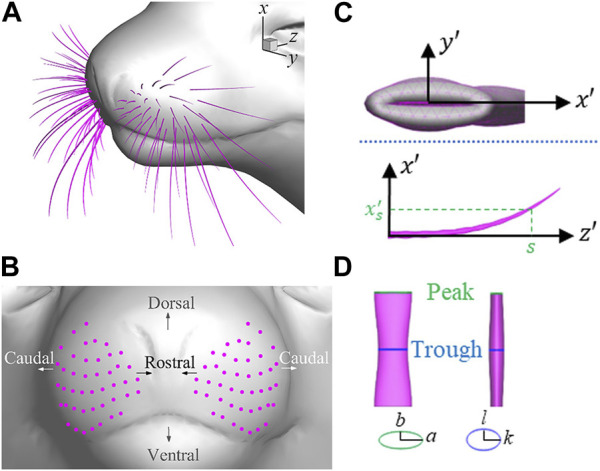
**(A)** Whisker array model of a harbor seal, **(B)** location of the base of each whisker, **(C)** Shape of an individual whisker, and **(D)** Side and front view of one wavelength segment of the seal whisker. The green and blue ellipses are the cross-section at the peak and the trough position, respectively.

The seal whisker array model, depicted in Figure 1A, is adopted from our previous study (Liu et al., 2021), which closely replicates a real harbor seal’s whisker array. The model comprises of two arrays, each containing 44 individual whiskers, placed on the muzzle of a realistic harbor seal head model. The length, curvature, tapering, location, and orientation of each whisker are taken from the reported data of realistic harbor seal whisker arrays (Murphy, 2013; Graff, 2016) and are illustrated in Figures 1B, C. The undulated geometry of each whisker is constructed using a simplified elliptical cylinder model, superimposed with two sinusoidal undulations with a 180° phase shift of the axes of the elliptical cross-sections (Figure 1D) (Rinehart et al., 2017). The material properties of the whiskers are also determined using experimental data (Hans et al., 2014). For more detailed information, please refer to Liu et al. (2021).

### 2.3 Simulation setup and parametric space

**FIGURE 2:**
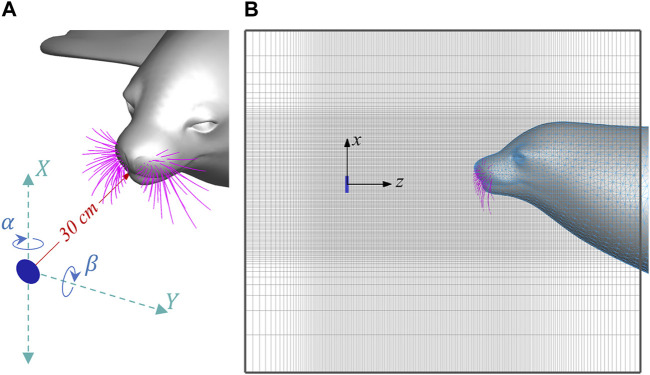
**(A)** Schematics of the upstream plate and X, Y, α and β parameters, and **(B)** Side view of the computational domain.

Figure 2 illustrates the simulation setup. A circular plate with a diameter of 40 mm is placed 30 cm in front of the nasal tip of a seal head. This diameter is determined based on previous studies (Wieskotten et al., 2011; Liu et al., 2021) for generating typical-size vortices of prey. The seal head and the plate are immersed into a 900 mm×1000 mm×950 mm flow domain. A uniform flow speed of 20 cm/s from left to right is applied as far field conditions on left, top, bottom, front and back boundaries of the flow domain and the outflow condition is implemented on the right boundary of flow domain with zero velocity gradient and zero pressure gradient. To reduce the computational time, we increased the kinematic viscosity of fluid to $4\times 10^{-5}m^{2}/s$ , leading to a reduced Reynolds number of ${Re}_{D}=200$ , defined using the diameter of the circular disk. To ensure grid independence, two different grids were tested, with resolutions of 128×128×256 and 256×256×256 grid cells in the *x*, *y*, and *z* directions, respectively. The highest grid density is concentrated near the circular disk, its wake region as well as the whisker arrays, with minimum grid cell sizes of 3.2 mm×4.4 mm×2.6 mm and 1.6 mm×2.2 mm×1.3 mm for the coarse and fine grids, respectively. The grid independence study shows that using the coarse grid results in less than 0.5% difference in the drag force experienced by the circular disk and 1% difference in the vortex shedding period compared to the fine grid. Considering the significant reduction of computational cost, the 128×128×256 coarse grid is chosen for the study, as depicted in Figure 2B. The flow simulations are executed on the XSEDE Expanse cluster (AMD EPYC 7742 type CPU, clock speed: 2.25 GHz, and flop speed: 4608 GFlop/s) using 16 processors. The simulations are run for 5,000 time steps to capture three steady vortex shedding cycles, resulting in an average computational cost of 224 CPU hours per case for the flow simulation.

The whiskers are discretized using ten-node tetrahedral cells, resulting in a range of 9,700–34,100 cells depending on the length of the whiskers. The whiskers are fixed at the base, preventing any movement or rotation. The solid simulations are performed on the same XSEDE Expanse cluster, with one processor allocated for each whisker. Thus, for each parametric case that includes 88 whiskers, the average computational cost for the solid simulation is 184 CPU hours.

A group of parametric simulations were conducted by systematically varying the location (X, Y) and orientation (α, β) of the circular plate, as defined in Figure 2A. The ranges of these parameters were determined to ensure the wakes would intersect at least one of the whiskers. The selected parameters are: X from −120 mm to 120 mm with an interval of 60 mm, Y from 0 to 200 mm with an interval of 50 mm, with an interval of 50 mm, α from −45° to 45° with an interval of 22.5°, and β from −45° to 45° with an interval of 22.5°. This results in a total of 625 simulation cases.

## 3 Interpretable deep learning network

### 3.1 Network architecture

Vision Transformer (ViT) (Vaswani et al., 2017; Dosovitskiy et al., 2020) has demonstrated effective structural interpretation of spatial correlations between pixels in images by dividing raw images into multiple image patches and leveraging comprehensive imaging information. Flexibility of ViT to handle various input sequences allows for the utilization of video information in addition to image information. In this study, we propose a network architecture called Video Transformer (VT) as a straightforward extension of ViT, designed specifically for video processing. VT initially splits the video into spatio-temporal tokens and applies a self-attention model using spatial and temporal blocks to extract features based on spatial and temporal changes of moments. These features are then optimized by passing through transformer blocks to determine the target position and orientation. In this section, we first introduce ViT, followed by a discussion on tubelet embedding for video processing (Arnab et al., 2021), and finally, we present the self-attention model for video processing (Arnab et al., 2021).

#### 3.1.1 ViT

Initially, ViT processes a 2D image, $I\in R^{H\times W\times C}$ , where $\left(H,W\right)$ represents the resolution of the original image, and C is the number of channels. The standard ViT extracts *M* non-overlapping image patches, $ΡATCH={\left.{\left\{patch\right.}_{i}\right\}}_{i=1}^{M}\in R^{M\times \left(h\times w\times c\right)}$ , where $\left(h,w\right)$ represents the resolution of each image patch, and c is the number of patch channels. The value of *M* is determined by $M=\left(H/h\right)\times \left(W/w\right)$ . These image patches are then flattened into 1D tokens, $Q={\left\{q_{i}\right\}}_{i=1}^{M}\in R^{M\times g}$ , where $g=\left(h\times w\times c\right)$ . These flattened tokens serve as the effective input sequence tokens for ViT. A constant latent vector size *G* is defined in ViT across all network layers, projecting the flattened 1D token g into a *G*-dimensional vector using a trainable linear projection. The output of this projection is considered as token embeddings. Similar to BERT’s token (Devlin et al., 2018), an optional learnable classification token $q_{class}$ is prepended to the sequence of embedded patches. Additionally, standard learnable 1D position embeddings $E_{pos}^{\prime }$ are added to the token embeddings to preserve positional information, which aids in learning the spatial relationship between whiskers. Finally, the sequence of token embeddings and position embeddings is passed through a transformer encoder (Vaswani et al., 2017), consisting of *L* transformer blocks. Each block l comprises alternating layers of layer normalization (LN), Multi-Headed Self-Attention (MSA), and Multi-Layer Perceptron (MLP), with a residual connection after MSA and MLP (Baevski and Auli, 2018; Wang et al., 2019). Therefore, the transformer encoder can be expressed as:

$$\begin{array}{l} Z_{0}=\left[q_{class};\;q_{1}E^{\prime };\;q_{2}E\prime ;\cdots ;q_{M}E\prime \right]+E_{pos}^{\prime },\\ E^{\prime }\in R^{g\times G},E_{pos}^{\prime }\in R^{\left(M+1\right)\times G},\; \end{array}$$

$${\dot{Z}}_{l}=MSA\left(\mathrm{LN}\left(Z_{l}\right)\right)+Z_{l},\;l=0\ldots 1,$$

$$Z_{l+1}=MLP\left(\mathrm{LN}\left({\dot{Z}}_{l}\right)\right)+{\dot{Z}}_{l},\;l=0\ldots 1,$$

where MLP consists of two layers with a GELU non-linearity (Hendrycks and Gimpel, 2016). Finally, an MLP is employed to classify the output of *L* transformer blocks, which can be expressed as:

$$target=MLP\left(\mathrm{LN}\left(Z_{L}\right)\right)$$

#### 3.1.2 Tubelet embedding

**FIGURE 3:**
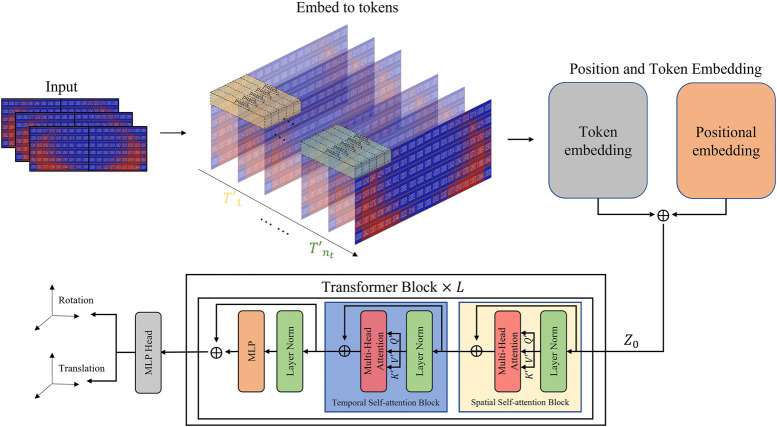
Video transformer network structure.

Given the flexibility of ViT to accommodate any sequence of input tokens $Z_{0}\in R^{M\times G}$ , we can leverage this property to convert image information into video information. For a video $V\in R^{T^{\prime }\times H\times W\times C}$ , where $T^{\prime }$ represents the temporal dimension, compared to image information $I\in R^{H\times W\times C}$ , the video data introduces an additional temporal dimension. Our goal is to map the video data to a sequence of tokens $Q^{v}\in R^{n_{t^{\prime }}\times n_{h}\times n_{w}\times g}$ . As depicted in “embed to token” in Figure 3, the process of tokenizing video information involves extracting non-overlapping spatio-temporal tubes from the input. These tubes are then projected to a sequence of tokens $Q^{v}\in R^{n_{t^{\prime }}\times n_{h}\times n_{w}\times g}$ , extending the embedding approach of ViT to a 3D setting.

In this context, we define a tubelet with dimensions $t^{\prime }\times h\times w$ , where tokens are derived from the temporal dimension $t^{\prime }$ and the spatial dimensions h and w . This allows us to obtain $n_{t^{\prime }}=\left\lfloor \frac{T^{\prime }}{t^{\prime }}\right\rfloor$ , $n_{h}=\left\lfloor \frac{H}{h}\right\rfloor$ , and $n_{w}=\left\lfloor \frac{W}{w}\right\rfloor$ frames. It is worth noting that smaller tubelets in any dimension result in an increase in computational cost during the training process, as more tokens are generated. Through tubelet embedding, the spatio-temporal information from video data can be directly fused into a transformer encoder, eliminating the need for the transformer to fuse temporal information from different frames.

#### 3.1.3 Factorized self-attention model

The factorized self-attention model (Ho et al., 2019; Weissenborn et al., 2019; Bertasius et al., 2021) described in the transformer block of Figure 3 processes the spatio-temporal tokens $Z_{0}$ obtained through the tubelet embedding method. Within each transformer block, the spatial self-attention block is initially applied to learn the spatial relationship among all tokens with the same temporal index, denoted as $T_{n_{i}}^{\prime }$ . Subsequently, the temporal self-attention block is employed to learn the temporal relationship among all tokens from the same spatial index, denoted as ${\left.{\left\{patch\right.}_{i}\right\}}_{i=1}^{M}$ . This enables effective learning of spatiotemporal interactions within each transformer block.

Specifically, the spatial self-attention block calculates the spatial relationship by reshaping the tokens $Z_{l}\in R^{n_{t^{\prime }}\times n_{h}\times n_{w}\times g}$ to $Z_{l}^{s^{\prime }}\in R^{n_{t^{\prime }}\times \left(n_{h}∙n_{w}∙g\right)}$ . Then, the output of the spatial self-attention block ${\dot{Z}}_{l}^{s^{\prime }}\in R^{n_{t^{\prime }}\times n_{h}\times n_{w}\times g}$ is reshaped to $Z_{l}^{t^{\prime }}\in R^{\left(n_{h}∙n_{w}\right)\times \left(n_{t^{\prime }}∙g\right)}$ and used as the input for the temporal self-attention block to calculate the temporal relationship. The factorized self-attention model can be summarized as follows:

$${\dot{Z}}_{l}^{s^{\prime }}=MSA\left(\mathrm{LN}\left(Z_{l}^{s^{\prime }}\right)\right)+Z_{l}^{s^{\prime }},\;l=0\ldots 1,$$

$${\ddot{Z}}_{l}^{t^{\prime }}=MSA\left(\mathrm{LN}\left(Z_{l}^{t^{\prime }}\right)\right)+Z_{l}^{t^{\prime }},\;l=0\ldots 1,$$

$$Z_{l+1}=MLP\left(\mathrm{LN}\left({\ddot{Z}}_{l}^{t^{\prime }}\right)\right)+{\ddot{Z}}_{l}^{t^{\prime }},\;l=0\ldots 1,\;$$

Unlike previous approaches, this model does not utilize a classification token since it can handle token reshaping across spatial and temporal dimensions without introducing ambiguities.

### 3.2 Network input preparation

The primary input signal for the network is derived from the time history of the bending moment at the base of each whisker. The total bending moment is decomposed in two components: $M_{x^{\prime }}$ and $M_{y^{\prime }}$ , where $x^{\prime }$ and $y^{\prime }$ denotes body-fixed coordinates of each whisker, as depicted in Figure 1C. Based on the direction of $x^{\prime }$ and $y^{\prime }$ , $M_{x^{\prime }}$ represents the bending moments resulting from the lift force while $M_{y^{\prime }}$ represents the bending moments resulting from the drag force. Please note that an additional simulation of the freestream passing the seal head without an upstream plate was conducted and the bending moments obtained from this simulation were then subtracted from the bending moments observed in the other cases where wakes were present. The process is to eliminate the influence of the freestream and solely examine the effect of the wakes.

Tactile vibrissal systems in general possess two distinct types of mechanoreceptors in their whiskers, namely Merkel cells (SAI mechanoreceptors) and Pacinian corpuscles (PC mechanoreceptors), specialized in detecting static and dynamic changes (Zimmerman et al., 2014). Merkel cells are adept at sensing static stimuli, such as sustained pressure, while Pacinian corpuscles excel at perceiving dynamic changes, including vibrations and rapid movements. To emulate the underlying nerve system, we further decompose the bending moments into two components: time-averaged (DC) components, denoted as $M_{DC}$ , and oscillatory components (AC), denotes as $M_{AC}$ . This yields a four-component vector: ( $M_{DC,x^{\prime }}$ ; $M_{AC,x^{\prime }}$ ; $M_{DC,y^{\prime }}$ ; $M_{AC,y^{\prime }}$ ), obtained from each whisker for the network input.

**FIGURE 4:**
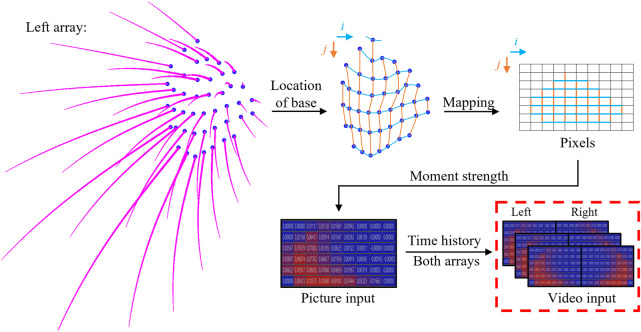
Data transformation process to generate the network signal map input from each individual whisker’s data.

The subsequent step involves a data transformation process aimed at consolidating the inputs from individual whiskers to generate a signal map, as depicted in Figure 4. Initially, the base locations of each whisker were extracted from the model and then topologically mapped onto a 9×22 two-dimensional rectangular grid based on their relative positions. Subsequently, each grid point was treated as a pixel and associated with the bending moment vector of the corresponding whisker. Each moment component was considered a distinct channel, with the intensity of each channel determined by the magnitude of its respective moment component. As a result, a “representative sensing image” comprising four channels was created. In cases where a pixel lacked correspondence to any whisker, a NULL (0) value was assigned. Given that bending moments exhibit temporal variations, the resulting data ultimately takes the form of a video file, reflecting the dynamic nature of the sensory input.

### 3.3 Training configuration

The network training process involves regression training for 1,000 epochs using the Adam optimizer. A batch size of 25 and an initial learning rate of 0.001 are used, along with a multi-step learning rate scheduler. The input samples are divided into train and validation groups using the 5-fold method, with 500 samples for training and 125 samples for validation. Each video sample has a size of 80×9×22×4 , representing the temporal dimension (80, corresponding to the last 1,500 flow time steps to only consider the steady vortices), the whisker array dimensions ( 9×22 ), and the moment channels (4). To capture the relationships between each whisker, the model parameters are initialized as follows: $t^{\prime }=8,h=1$ and w= 1, with L=4 and *G* = 96.

The implementation of the method utilizes PyTorch 1.12.1 and Skorch 0.11.0 (Tietz et al., 2017) frameworks. The training process is performed using an NVIDIA GeForce RTX 2080 Ti GPU with 11GB memory.

## 4 Results and discussion

This section begins with a comprehensive examination of the influence of the plate’s location and orientation on both the flow field and whisker signals. Subsequently, the outcomes of the interpretable deep learning network are presented, including accuracy measurements and importance value maps of the signals. Lastly, the correlation between the importance value maps, whisker signals, and flow field is analyzed, while also investigating the underlying mechanisms. To facilitate the discussion, five exemplary cases are selected for a detailed investigation, which are summarized in Table 1.

**TABLE 1:** Definition of the exemplary cases.

Case	X	Y	α	β
$X_{60}$	60 mm	0	0	0
$Y_{50}$	0	50 mm	0	0
$\alpha_{22.5}$	0	0	22.5°	0
$\beta_{22.5}$	0	0	0	22.5°
$\alpha_{45}\beta_{22.5}$	0	0	45°	22.5°

### 4.1 Characteristics of the flow field

**FIGURE 5:**
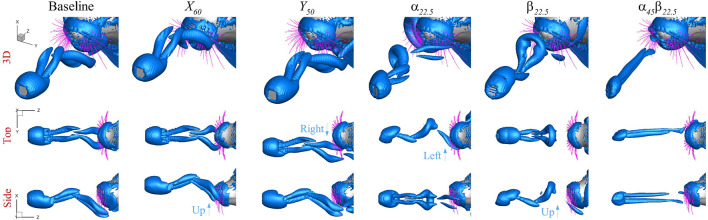
Plate induced wake structure determined by the $\lambda_{2}$ criterion from viewpoints of 3D, top view, and side view, in the baseline, $X_{60}$ , $Y_{50}$ , $\alpha_{22.5}$ , $\beta_{22.5}$ and $\alpha_{45}\beta_{22.5}$ cases.

Figure 5 shows the wake structure induced by the plate in the exemplary cases, determined by the $\lambda_{2}$ criterion, viewed from different viewpoints: 3D, top view, and side view. To aid the comparison, the baseline case is added in the figure. Except for $\alpha_{45}\beta_{22.5}$ case, the wake displays two distinct vortex rings that alternate between the upper and lower sides ( $X_{60}$ , $Y_{50}$ , and $\beta_{22.5}$ cases) or the left and right sides ( $\alpha_{22.5}$ case), with a phase difference of 180°. In the baseline case, where the plate is directly in front of the seal’s nose, the wake interacts with all whiskers. As the plate moves upward in the $X_{60}$ case and rightward in the $Y_{50}$ case, the wake translates accordingly without changing the pattern. In the $\alpha_{22.5}$ case, where the plate tilts to the left, the wake inclines in the same direction. Additionally, due to plate rotation, the vortex ring forming on the left side is stronger than the one on the right side. In the $\beta_{22.5}$ case, where the plate tilts upward, the wake also inclines upward accordingly, and the rotation generates a stronger vortex ring on the upper side than the lower side. In contrast, the $\alpha_{45}\beta_{22.5}$ case does not exhibit vortex shedding but instead displays two streamwise vortex legs attached to the plate’s sides.

**FIGURE 6:**
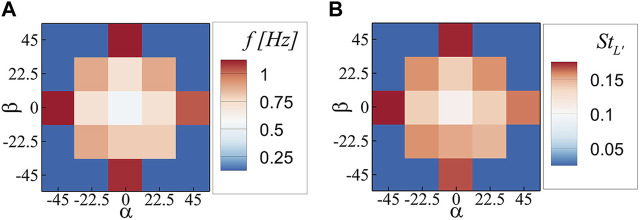
**(A)** Vortex shedding frequency, and **(B)** Strouhal number; in the parametric space of α and β.

The vortex shedding frequency also changes with the orientation of the plate. To illustrate the relationship, Figure 6A presents the vortex shedding frequency in the parametric space of α and β. When vortex shedding is absent, a zero value is assigned. The plot reveals that the vortex shedding frequency increases symmetrically along both the α and β axes. When one orientation angle is at its maximum while the other is non-zero, no vortex shedding occurs. To account for the changes in both the vortex shedding frequency and the frontal area of the plate, the Strouhal number was calculated for each case using the formula ${St}_{L^{\prime }}=fL^{\prime }/U_{\infty }$ , where f represents the vortex shedding frequency, $U_{\infty }$ denotes the inflow velocity, and L ′ is the hydraulic diameter of the frontal area of the plate. Figure 6B demonstrates the change in the Strouhal number with orientation, mirroring the behavior of the vortex shedding frequency. The maximum increase in the Strouhal number is 80%. A similar observation was reported by Chen and Fang (1996), where a decrease in the angle of attack of an inclined plate (comparable to increasing α and/or β in our study) resulted in an increase in the Strouhal number of its vortex shedding.

### 4.2 Whisker signals

**FIGURE 7:**
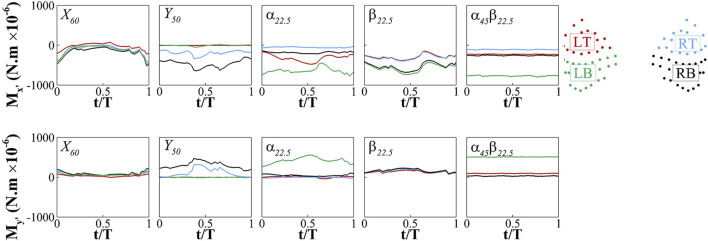
Phase-averaged time history of $M_{x^{\prime }}$ and $M_{y^{\prime }}$ averaged over the LT (left top), LB (left bottom), RT (right top), and RB (right bottom) whiskers for $X_{60}$ , $Y_{50}$ , $\alpha_{22.5}$ , $\beta_{22.5}$ and $\alpha_{45}\beta_{22.5}$ cases.

We partition the whisker arrays into four regions: LT (left top), LB (left bottom), RT (right top), and RB (right bottom) whiskers, and calculated the average signals, $M_{x^{\prime }}$ and $M_{y^{\prime }}$ , across the whiskers within each region. Figure 7 presents the phase-averaged time history of $M_{x^{\prime }}$ and $M_{y^{\prime }}$ in each region in each exemplary case. Note that $M_{x^{\prime }}$ is mostly negative values in all the cases, while $M_{y^{\prime }}$ exhibits both positive and negative values. The behaviour of the signals aligns with the temporal and spatial dynamics of the vortices in each case, (e.g. Frequency). In the $X_{60}$ case, the signals are evenly distributed across the regions since the vortices impact the seal head at the center. In the $Y_{50}$ case, where the wake translates rightward, both $M_{x^{\prime }}$ and $M_{y^{\prime }}$ are stronger on the right whiskers. The right bottom whiskers experience stronger signals compared to the right top whiskers because of the impact location of the vortices. In the $\alpha_{22.5}$ case, the left whiskers experience stronger signals than the right whiskers, consistent with the plate rotation that generates stronger vortices on the left side. Additionally, the left bottom whiskers experience stronger moments than the left top whiskers. In the $\beta_{22.5}$ case, interestingly, despite the upper vortex ring being stronger than the bottom one, the bottom whiskers experience stronger $M_{x^{\prime }}$ than the top whiskers. This is attributed to the upward inclination of the wake, causing the upper vortex ring moving away from the whiskers. Also interestingly, different from $M_{x^{\prime }}$ , $M_{y^{\prime }}$ shows no variation across the regions in this case. In the $\alpha_{45}\beta_{22.5}$ cases, both $M_{x^{\prime }}$ and $M_{y^{\prime }}$ are almost constant with time while the left bottom whiskers have stronger moments compared to the other whiskers due to the stronger wake in this region.

Overall, the signals at the root of the whisker arrays exhibit temporal and spatial patterns that align with the dynamics of the vortices. There is a clear correlation between the arrival of the vortices and the temporal variation in the signals. The regions where the vortices directly impact the whiskers tend to exhibit stronger signal amplitudes. The distinct vortex dynamics in each case result in different temporal and spatial patterns of the signals, providing an explanation for the discernible differences among the cases.

### 4.3 Network results

**FIGURE 8:**
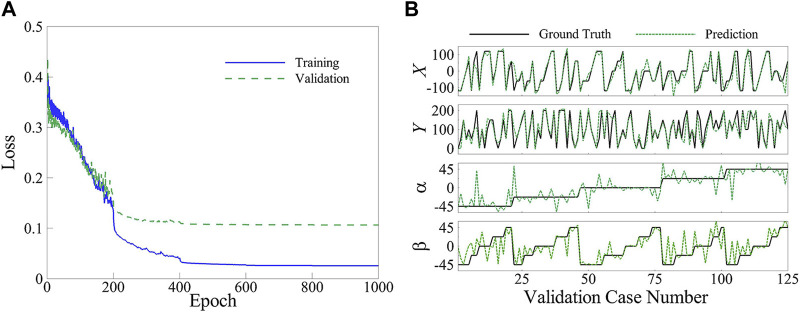
**(A)** Mean squared error loss of the training and validation groups, and **(B)** Ground truth and predicted values for the validation group.

The neural network was trained successfully to predict the location and orientation of the upstream plate. Figure 8A illustrates the best mean squared error (MSE) losses of the training and validation groups over 1,000 epochs, indicating the convergence of the network. The final MSE loss for the training and validation corresponding to the best fold is 0.0255 and 0.1064, respectively. The decision to limit the epochs to 1,000 was based on the observation that the drop in MSE loss for both groups during the last 100 epochs is less than 0.1%. The accuracy of the trained network corresponding to the best fold is depicted in Figure 8B through a comparison of the ground truth and predicted locations and orientations of the upstream plate in the validation cases. The accuracy metric is quantified using the following formula:

$$Accuarcy=\left(1-\frac{\sum \left(\frac{\left|y_{GT}-y_{\mathrm{Pr}}\right|}{Range}\right)}{N}\right)\times 100$$

where $y_{GT}$ represents the ground truth value, $y_{\mathrm{Pr}}$ denotes the predicted value, Range represents the range of changes of $y_{GT}$ , and N is the number of validation cases. The obtained 5-fold-average accuracy of the parameters X , Y , α , and β is 91.4±1.1% , 91.0±1.1% , 84.8±2.6% , and 83.3±0.6% , respectively, resulting in an average accuracy of 87.6±0.7% for the network.

An essential capability of the interpretable network is its ability to generate temporal and spatial importance of the signals within the trained network, allowing for the identification of significant temporal-spatial signal patterns that contribute to the network’s predictions. In our study, as outlined in Section 3.3, we utilized a temporal dimension of 80 and a time interval of t = 8, resulting in 10 distinct time patches for the prediction. In the $X_{60}$ and $Y_{50}$ cases, these 10 time patches correspond to two consecutive cycles, while in the $\alpha_{22.5}$ and $\beta_{22.5}$ cases, they correspond to three consecutive cycles.

**FIGURE 9:**
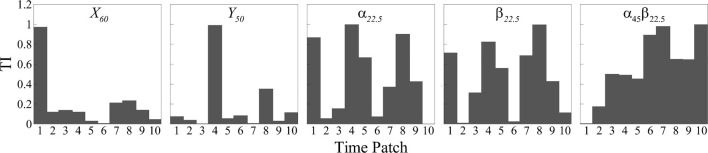
Temporal importance map of $X_{60}$ , $Y_{50}$ , $\alpha_{22.5}$ , $\beta_{22.5}$ and $\alpha_{45}\beta_{22.5}$ cases in the trained network.

In Figure 9, we present the temporal importance (TI) analysis of the signals in each representative case. Except for the $\alpha_{45}\beta_{22.5}$ case, the results highlight that the temporal importance is not uniformly distributed across all time instances. Instead, certain time points exhibit significantly higher importance values, while others demonstrate relatively lower importance values. This disparity suggests that specific time instances play a crucial role in the prediction process, while others have a lesser impact. Furthermore, it is noteworthy that the temporal importance patterns differ across all the cases. Each case exhibits a unique distribution of importance values across the time patches. In the $\alpha_{45}\beta_{22.5}$ case where vortex shedding is absent, the temporal importance is nearly uniform acorss the time patches within a cycle. It is because the flow field remains nearly unchanged with time.

**FIGURE 10:**
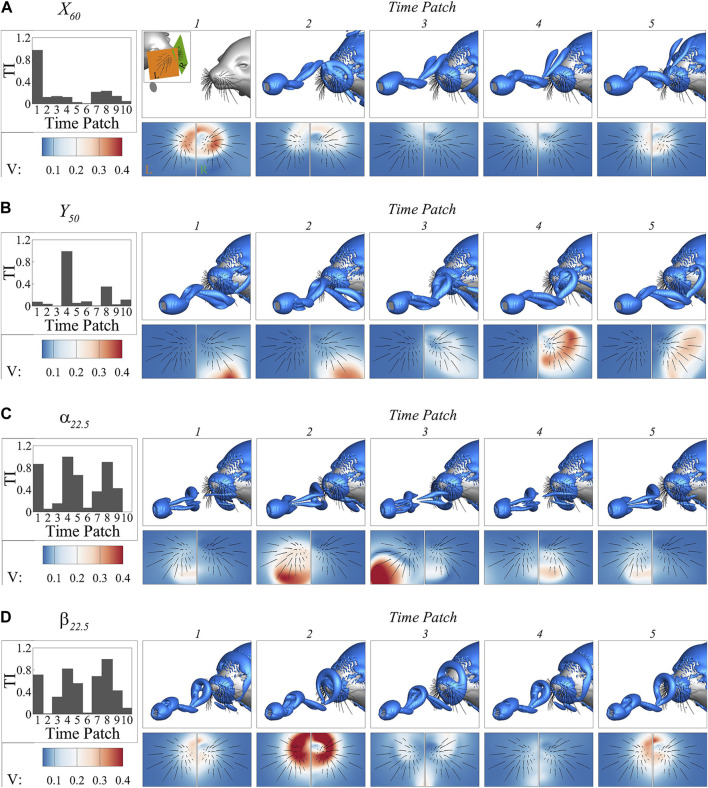
The vortex structures determined by the $\lambda_{2}$ criterion and contours of flow velocity magnitude at two planes intersecting the whisker arrays at the first five individual time patches of **(A)** $X_{60}$ , **(B)** $Y_{50}$ , **(C)** $\alpha_{22.5}$ , and **(D)** $\beta_{22.5}$ cases. Note that the flow velocity in the case of freestream passing the seal head without an upstream plate was subtracted from the flow velocity in other cases where wakes were present to focus on the disturbance induced by the wake.

The variation in temporal importance patterns highlights the importance of considering the temporal dynamics and their specific correlations with the underlying flow structures when interpreting the prediction process. To illustrate this relationship, we present the vortex structures determined by the $\lambda_{2}$ criterion and contours of flow velocity magnitude at two planes intersecting the whisker arrays at the first five individual time patches in each representative case in Figure 10. Note that the flow velocity in the case of freestream passing the seal head without an upstream plate was subtracted from the flow velocity in other cases where wakes were present to focus on the disturbance induced by the wake. To facilitate analysis, the temporal importance map is also included in each case. These five patches cover either one or more than one cycle of the wake. The $\alpha_{45}\beta_{22.5}$ case is not included as the temporal importance and flow structure remains unchanged in this case.

In the $X_{60}$ case, time patch 1 exhibits significantly higher importance compared to the other time patches. This observation corresponds to the vortex dynamics, where at time patch 1, the lower vortex ring actively interacts with the whiskers. In contrast, at the other time patches, either the top vortex ring arrives in the whisker area but lacks strong interaction due to its distance from the whiskers, or the bottom vortex ring has already moved past the whiskers. The flow velocity contour reveals that the flow disturbance is strongest at time patch 1 around the whisker compared to other time patches.

In the $Y_{50}$ case, time patch 4 demonstrates significantly higher importance compared to other time patches. This time patch corresponds to the instances when the top vortex ring interacts with the right whiskers. At the other time patches, either the top vortex ring has not yet reached the whiskers or has already moved past them. In this case, the bottom vortex ring has minimal effect due to its distance from the whiskers resulting from the translation of the upstream plate. The flow velocity contour also shows that the vortex ring interacts with the right whisker array and the disturbance is the strongest on most whiskers at time patch 4.

In the case of $\alpha_{22.5}$ time patch 4 exhibits the highest importance, while time patches 1 and 5 are slightly less important, and time patches 2 and 3 have minimal importance. Interestingly, upon analyzing the vortex dynamics, it is observed that time patches 2 and 3 capture the interactions between the left whiskers and the stronger vortex ring from the left side. Conversely, time patch 4 captures the interactions between the right whiskers and the weaker vortex ring shed from the right edge. Time patches 1 and 5 represent the transitional stages, where the weaker vortex ring is about to leave and the stronger vortex ring is about to arrive. The flow velocity contour further confirms that the flow disturbance is strongest at time patches 2 and 3 due to the presence of the stronger vortex, whereas it is weaker at time patch 4 attributed to the weaker vortex ring. One possible explanation for the higher importance of the weaker vortex in this case is that the stronger vortex ring shed from the left edge aligns towards the center of the nose, similar to the vortices observed in other cases. In contrast, the weaker vortex ring is oriented perpendicular to it and only interacts with the right whisker arrays. This unique interaction provides additional crucial orientation information, making the weaker vortex more influential in the prediction process.

In the case of $\beta_{22.5}$ , time patches 1, 4, and 5 exhibit greater significance compared to time patches 2 and 3. Through vortex dynamics analysis, it is observed that time patches 1 and 5 capture the arrival of the top vortex at the whiskers, while time patch 4 captures the interactions between the bottom vortex and the whiskers. Interestingly, time patch 2 captures the strong interactions between the top vortex ring and the whiskers, but its contribution is nearly negligible. This observation is further supported by the flow velocity contour. The flow disturbance during time patches 1, 2, and 5 is primarily associated with the top vortex, whereas during time patch 4, it is linked to the bottom vortex. Time patch 3 presents a scenario where disturbances from both vortex rings are present. Notably, similar to the $\alpha_{22.5}$ case, the two vortex rings differ in strength, with the top ring being stronger than the bottom one, but the weaker ring plays the most important role in prediction. It is possible for the same reason that the weaker ring possesses a unique orientation and only interacts with the bottom whiskers. However, unlike the $\alpha_{22.5}$ case, the significance of the other ring is increased in this case. It may be because the orientation information provided by the bottom ring is insufficient for accurate prediction, thereby necessitating the incorporation of additional information.

**FIGURE 11:**
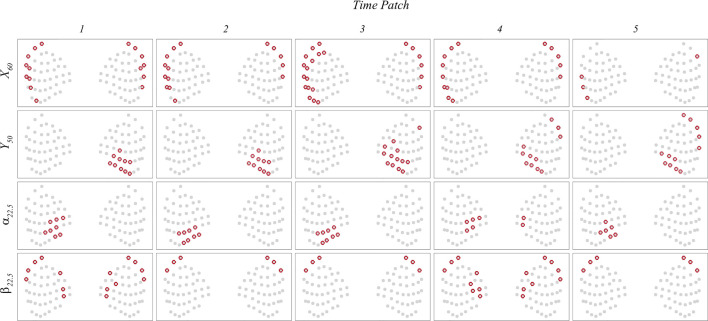
The locations of the whiskers with the highest importance values during each of the first five time patches in $X_{60}$ , $Y_{50}$ , $\alpha_{22.5}\;and$ $\beta_{22.5}$ cases.

The current interpretable deep learning network also provides the importance of each whisker through spatial importance (SI) maps at individual time patch. Figure 11 illustrates the locations of the whiskers with the highest importance values during each of the first five time patches in each case. These whisker locations generally exhibit a strong correlation with areas of strong flow disturbance, as depicted in Figure 10. Furthermore, the spatial distribution of these important whiskers provides insights into the location and size of the vortex rings in the wake. For instance, in the $X_{60}$ case, the important whiskers reflect the vortex ring impacting the center of the seal head. In the $Y_{50}$ case, the important whiskers correlate with strong flow disturbance on the right side, and their extension reflects the extension of the vortex ring. In the $\alpha_{22.5}$ case, the important whiskers capture both vortex rings, with one located on the left side and the other on the right side. Also, they reveal that the extension of the left vortex ring is larger than the right one. Similarly, in the $\beta_{22.5}$ case, the important whiskers capture two vortex rings. In this scenario, they capture that the higher vortex ring is larger and situated near the center, while the lower vortex ring is smaller and positioned closer to the bottom.

**FIGURE 12:**
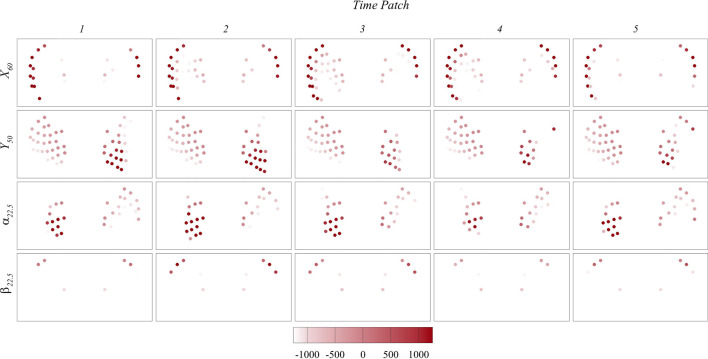
Contour maps of $M_{x^{\prime }}$ on the whisker arrays during each time patch in $X_{60}$ , $Y_{50}$ , $\alpha_{22.5}\;and$ $\beta_{22.5}$ cases.

**FIGURE 13:**
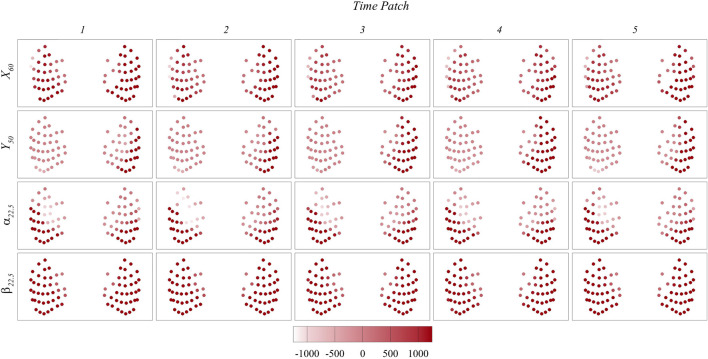
Contour maps of $M_{y^{\prime }}$ on the whisker arrays during each time patch in $X_{60}$ , $Y_{50}$ , $\alpha_{22.5}\;and$ $\beta_{22.5}$ cases.

To gain a deeper understanding of the signals used for prediction, Figures 12, 13 present the maps of $M_{x^{\prime }}$ and $M_{y^{\prime }}$ on the whisker arrays during each time patch for each case. Note that $M_{x^{\prime }}$ represents the moment generated by the lift force, while $M_{y^{\prime }}$ represents the moment generated by the drag force. A strong correlation between the important whiskers and $M_{x^{\prime }}$ maps is observed across all cases, indicating the significant role of $M_{x^{\prime }}$ in the prediction process. Conversely, we did not observe any notable correlation with the $M_{y^{\prime }}$ maps, suggesting that $M_{y^{\prime }}$ has minimal impact on the prediction. Interestingly, the direction of $M_{x^{\prime }}$ plays a crucial role in the prediction, as the important whiskers consistently correlate with positive $M_{x^{\prime }}$ values across all cases or, in cases where positive $M_{x^{\prime }}$ values are absent, with less negative $M_{x^{\prime }}$ values. It is important to note that in our algorithm, the calculation of $M_{x^{\prime }}$ involves subtracting the moment induced by the freestream flow. This subtraction is intended to focus on the moment induced specifically by vortices. Further studies are needed to clarify whether the direction information remains important if the original moment, without subtracting the freestream moment, is used. Nevertheless, the results suggest that the bending moment generated by the lift force plays a crucial role in the prediction process, and the direction of this moment may also be important.

## 5 Summary and conclusion

In this study, we developed a novel method that combines a computational fluid-structure interaction (FSI) model with interpretable deep-learning models to investigate the fundamental mechanisms of seal whisker sensing. We demonstrate that the method is able to accurately predict the location and orientation of a circular plate upstream of a seal’s head by using the bending moment signals at the root of whisker arrays. The algorithm also reveals crucial temporal-spatial signal patterns utilized in the prediction process, enabling establishing correlations between important signal patterns, flow features, and characteristics of upstream obstacles for enhanced understanding.

The FSI model couples an incompressible flow solver and structural dynamics solver in a one-way coupling manner. We generated diverse wake characteristics and corresponding whisker dynamics by placing a circular plate upstream of a seal’s head and systematically varying the location and orientation of the plate. Two types of wake patterns were observed. For most of the cases, the wake is characterized by two vortex rings shed from two edges of the plate with a phase difference. The translation of the upstream plate solely affects the position of the wake, without altering its structures. However, when the orientation of the plate is changed, it induces an inclination in the wake’s trajectory, a shift in the orientation of the vortices, and an increase of the vortex shedding frequency. Furthermore, the side farther from the inflow corresponds to the location of the stronger vortex, while the side closer to the inflow is associated with the weaker vortex. In contrast, for the cases where one of the orientation angles is the largest while the other is non-zero, no vortex shedding occurs in the wake. Instead, the wake is characterized by two streamwise vortex legs attached to the side of the plate. The whisker signals are represented by the bending moments at the root of each whisker. The signals exhibit temporal and spatial patterns that align with the dynamics of the vortices. There is a clear correlation between the arrival of the vortices and the temporal variation in the signals. The regions where the vortices directly impact the whiskers tend to exhibit stronger signal amplitudes.

We train the network model to learn the location and orientation of the upstream plate based on the whisker signals. The results show that the model is able to predict the location of the plate with the accuracy of 90%–92.0% and the orientation of the plate with the accuracy between 86% and 89%. The analysis on the temporal importance of the signals reveals that the prediction process primarily relies on the time instances when the wake vortices actively interact with the whiskers. When the vortices are at varying distances from the whiskers, such as when the upstream plate is translated, interactions with the vortices in close proximity to the whiskers have a more significant impact, while interactions with the distant vortex ring have minimal influence. In the context of predicting the orientation of the upstream plate, interactions with all vortices can be important. However, interactions involving the vortices that carry more orientation information tend to hold greater significance, even if these vortices do not cause the strongest disturbance and signals. It suggests that the vortices that provide crucial orientation cues play a crucial role in the prediction process, even if their influence on the overall flow disturbance and signal strength is not the most pronounced. Spatially, the whiskers in stronger flow disturbance areas are more important in the prediction. The spatial arrangement of the important whiskers not only captures the vortex ring structure in the wake, but also provides valuable information about the location and size of the rings. In the context of the signals used for prediction, our results suggest that the bending moment generated by the lift force plays a crucial role in the prediction process. The direction of the moment may also be important as important whiskers are all associated with the bending moment in one direction.

Taken together, we demonstrate that the developed interpretable neural network is able to accurately predict the location and orientation of the upstream object of seal whisker arrays. More importantly, it allows for the identification of significant temporal-spatial signal patterns that contribute to the network’s predictions, which can then be further correlated with flow structures, enabling the identification of the crucial flow features that are sensed for accurate prediction. These insights are crucial for the development of intelligent flow sensors capable of accurately perceiving and interpreting complex underwater environments.

We would like to acknowledge that our study utilized a simplified drag-lift model in our one-way coupling approach to estimate the hydrodynamic loadings along each whisker. This simplified model does not take into account the feedback effect of the whiskers on the surrounding flow. In reality, the presence of the whiskers would influence the surrounding flow, resulting in different drag and lift values that could potentially affect the outcomes of our research. However, we believe that the impact of the whiskers on the surrounding flow would be small due to the small diameter of the whiskers (with a mean diameter of 0.66 mm). This diameter is approximately 1-2 orders of magnitude smaller than the scale of the vortices in the wakes, which range from 20 mm to 120 mm. Considering this scale difference, we believe that the one-way coupling approach still provides valuable insights while significantly reducing the computational cost associated with conducting full FSI simulations. It is important to note that future investigations could explore the implications of the two-way coupling between the whiskers and the surrounding flow for a more comprehensive understanding of the hydrodynamic interactions.

In all the simulation cases, we observed that the frequency of the bending moments consistently matched the shedding frequency of the wake vortices. This behavior is a direct consequence of the one-way coupling approach, where the feedback effect of whisker dynamics on the flow was disregarded. It is worth noting, however, that in our previous research (Liu et al., 2019b), we discovered that the distinctive undulated geometry of seal whiskers can effectively mitigate vortex-induced vibration when exposed to steady flows. This unique characteristic enables the whiskers to better synchronize with the wake frequency. Nonetheless, it is important to acknowledge that further investigations are required to explore the interactions between vortex-induced vibration and wake-induced vibration. Understanding these complex dynamics will provide valuable insights into the combined effects and enhance our comprehension of the overall system behavior.

Finally, seal whisker arrays display distinct spatial grid patterns combined with variations in whisker length and orientation. These patterns are believed to play crucial functional roles in enhancing the seals’ sensing capabilities. For instance, it is believed that longer whiskers located on the caudal side of the array can provide a larger detection area, extending the reach and sensitivity to water movements. Moreover, the variation in whisker length and orientation may allow for high sensitivity in wide frequency range and flow direction. Further studies are needed to fully investigate these effects, particularly by comparing the sensing capabilities between different whisker arrangements. The current findings are limited to a single configuration, and more research is necessary to gain a comprehensive understanding.

## Data Availability

The raw data supporting the conclusion of this article will be made available by the authors, without undue reservation.
